# Risk factors and quality of life in adults with dysphagia after posterior skull base surgery: a cross-sectional study

**DOI:** 10.3389/fonc.2025.1582176

**Published:** 2025-08-29

**Authors:** Qiao Wang, Shang-Jin Cheng, Dan Duan, Wen-Yao Cui, Wen-Jie Liu

**Affiliations:** Department of Neurosurgery, West China Hospital, Sichuan University/West China School of Nursing, Sichuan University, Chengdu, China

**Keywords:** posterior fossa tumor, surgery, dysphagia, quality of life, influencing factors

## Abstract

**Introduction:**

The study aimed to evaluate the current status of swallowing function and quality of life in patients undergoing posterior skull base surgery, identify risk factors for dysphagia, and provide evidence for early interventions.

**Method:**

Patients undergoing posterior skull base surgery were prospectively enrolled, from June 2023 to June 2024. Data collection included demographics, disease-related details, swallowing function assessment, and scores of Swallowing-Quality of Life (SWAL-QOL). The logistic regression was used to identify risk factors influencing dysphagia, while the SWAL-QOL questionnaire was used to investigate the quality of life in patients undergoing posterior skull base surgery.

**Results:**

Among the 143 patients, approximately 50% developed postoperative dysphagia. Logistic regression analysis identified a history of choking, surgical duration, and the total score of nutritional risk as independent predictors of dysphagia. The area under the Receiver Operating Characteristic Curve was 0.797 (95%CI: 0.722–0.871). The SWAL-QOL questionnaire revealed significantly lower scores across all dimensions in patients with dysphagia (*P* < 0.05), particularly in swallowing function, eating duration, and food selection.

**Conclusion:**

Postoperative dysphagia is prevalent after posterior skull base surgery and significantly impairs quality of life. Routine screening and proactive management of swallowing function are essential for improving clinical outcomes and enhancing swallowing-related quality of life.

## 1 Introduction

The posterior skull base houses critical and complex structures, including the cerebellum and brainstem, which play a pivotal role in executing precise and efficient swallowing functions (1, 2). Surgical intervention remains the preferred treatment for skull base tumors. However, the posterior skull base is characterized by its limited space, deep location, and close anatomical relationship with the posterior cranial nerves—namely, the glossopharyngeal, vagus, parasympathetic, and hypoglossal nerves—that regulate swallowing function. Consequently, both the tumor itself and surgical procedures can damage or strain these nerves, leading to severe complications such as muscular weakness, sensory alterations, imbalance, cognitive dysfunction, and dysphagia, with dysphagia being particularly common after surgery in this region. Additional factors, such as endotracheal intubation, tracheotomy, and postoperative swelling or compression of cranial nerves, further increase the risk of postoperative swallowing disorders (3).

Dysphagia, defined as impaired transport of food from the mouth to the stomach due to structural or functional deficits, is a major cause of aspiration pneumonia. This condition, associated with disrupted swallowing mechanics and inadequate airway protection, often necessitates prolonged hospitalization and can result in patient mortality (4). Moreover, dysphagia can lead to severe complications, including dehydration, malnutrition, and psychological distress, significantly impairing physical and mental health and diminishing quality of life (5–7). Given its substantial impact on postoperative outcomes in patients undergoing posterior skull base surgery, early recognition and assessment of dysphagia are critical. Timely detection allows for interventions such as compensatory strategies (8), dietary modifications, and swallowing therapy (9), mitigating its functional impact (10). Advances in medical technology have improved survival rates following posterior skull base surgery, heightening interest in strategies to manage postoperative dysphagia and enhance functional outcomes (7, 11). However, research on postoperative dysphagia has predominantly focused on pediatric populations (12, 13). This imbalance suggests that adult dysphagia may be underrecognized, and pediatric-based risk models might not generalize to adults. Our study addresses this gap by focusing on age-specific risk profiles and the impacts on quality of life.

## 2 Materials and methods

### 2.1 Ethics approval and consent to participate

This cross-sectional study was conducted in compliance with the ethical principles outlined in the Declaration of Helsinki and approved by the Biomedical Ethics Review Committee of West China Hospital, Sichuan University (No. 20241935). All respondents agreed to participate in this study.

### 2.2 Participants and procedure

**Figure 1:**
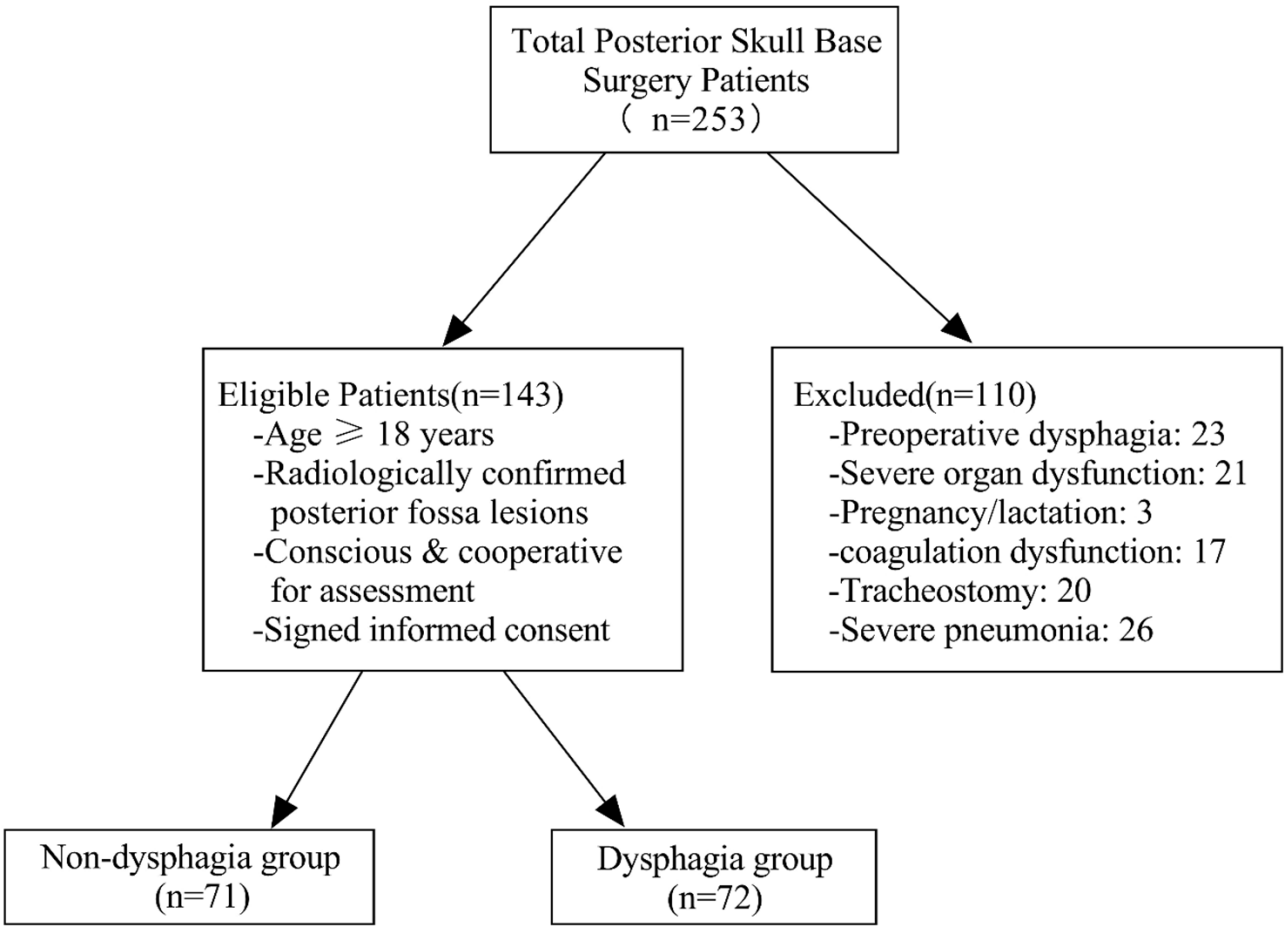
Patient screening flowchart.

Patients undergoing posterior skull base surgery were recruited from June 2023 to June 2024 in the neurosurgery department of the hospital. Inclusion criteria were as follows: (1) patients aged 18 years old and older; (2) CT or MRI showed posterior fossa occupation (e.g., tumors in the jugular foramen, rocky oblique region, pontine cerebellar angle, acoustic neuromas, or foramen magnum of the occipital bone); (3) patients undergoing posterior skull base surgery with sufficient consciousness and ability to cooperate in swallowing function assessments; and (4) informed consent for voluntary participation. Exclusion criteria were as follows: (1) Dysphagia was present before posterior skull base surgery; (2) severe liver, kidney, or cardiac abnormalities; (3) pregnancy or lactation; (4) coagulation dysfunction; (5) tracheotomy; and (6) severe pneumonia. Details are shown in **Figure 1**.

### 2.3 Observation indicators

#### 2.3.1 Demographic and disease-related information

Demographic and disease-related information of enrolled patients were collected. Demographic data included age, gender, education, marital status, occupation, smoking history, and alcohol consumption history. Disease-related data included comorbidities, dentures, history of choking, surgical site, surgical duration, anesthesia duration, presence of gastric tube, hemoglobin, serum albumin, total cholesterol, triglyceride, and the Nutritional Risk Score 2002 (NRS 2002).

#### 2.3.2 Swallowing function assessment

The Eating Assessment Tool-10 (EAT-10) developed by Belafsky et al. in 2008 was used to screen for dysphagia in high-risk populations (14). Zeng and colleagues conducted culturally adaption and verification on the Chinese version of EAT-10 (15). The tool consists of 10 questions addressing dysphagia symptoms, clinical features, psychological impacts, and social consequences. Responses are scored on a 5-point scale, with a total score of more than 3 points indicating dysphagia. The content validity index was 0.95 for the scale and >= 0.8 for each item (15). A recent systematic review reported its sensitivity and specificity for dysphagia screening at 0.85 (95% CI: 0.68–0.94) and 0.82 (95% CI: 0.65–0.92), respectively (16).

#### 2.3.3 Swallowing-quality of life questionnaire

The Swallowing-Quality of Life (SWAL-QOL) questionnaire is a self-assessment tool designed by McHorney et al. in 2002 for patients with swallowing disorders of various causes (17). The questionnaire includes 11 dimensions and 44 items, with eight dimensions focusing on dysphagia-related quality of life (e.g., swallowing burden, eating duration, eating desire, food selection, fear of intake, communication, and mental health, social functioning), two addressing general quality of life (fatigue and sleep), and one evaluating swallowing symptom frequency through 14 items. Responses are scored on a 5-point Likert scale and converted to a 0–100 metric, with higher scores reflecting better.

#### 2.3.4 Data collection

On postoperative day one (POD1), after regaining consciousness, the neurosurgical team promptly administered the Modified Water Swallow Test (MWST) to screen for aspiration risk. Patients who passed the MWST were cleared for oral feeding, whereas those with suspected aspiration were referred to the rehabilitation department for further assessment. Within 24 hours of completing the MWST, neurosurgery and rehabilitation physicians jointly conducted the EAT-10 to stratify patients into dysphagia (EAT-10 ≥ 3) and non-dysphagia (EAT-10 < 3) groups. To evaluate the acute-phase impact of dysphagia on quality of life, all patients subsequently completed the SWAL-QOL questionnaire immediately after the EAT-10 assessment. Finally, demographic and disease-related information of enrolled patients were retrieved from the hospital’s electronic medical record system to complete the dataset. This standardized protocol, implemented by a multidisciplinary team (physicians, swallowing therapists, nursing supervisors, and trained nurses), ensured all assessments were completed within the critical 24-to-48-hour postoperative window while maintaining methodological rigor.

### 2.4 Statistical analysis

All statistical analyses were conducted using SPSS version 22.0 (IBM Corp., Armonk, NY, USA). Continuous variables with a normal distribution were expressed as mean ± standard deviation (SD) and compared using independent-samples t-tests. Non-normally distributed data were reported as median (interquartile range, IQR) and analyzed using the Mann–Whitney U test. Categorical variables were summarized as frequencies (percentages), and group comparisons were performed using Pearson’s chi-square test or Fisher’s exact test, as appropriate.

Potential risk factors for dysphagia were initially identified through univariate analysis (*P* < 0.05). Multicollinearity among candidate variables was assessed using variance inflation factors (VIF), with variables exhibiting VIF ≥ 5 excluded from further analysis. Independent risk factors were subsequently identified via binary logistic regression. Model calibration was evaluated using the Hosmer–Lemeshow goodness-of-fit test, while model discrimination was assessed using receiver operating characteristic (ROC) curve analysis.

## 3 Results

### 3.1 Demographic and disease-related characteristics

A total of 143 patients undergoing posterior skull base surgery were included in the final analysis. No patients were lost to follow-up or excluded after the initial enrollment phase. According to EAT-10 questionnaire, 71 patients did not have dysphagia, while 72 (approximately 50%) had dysphagia. The cohort consisted of 63.6% females and 36.4% males. Dysphagia prevalence was higher in males (63.46%) compared to females (42.86%). The NRS 2002 indicated significantly higher nutritional risk in the dysphagia group than in the non-dysphagia group (*P* < 0.05). Detailed demographic and disease-related characteristics are presented in **Table 1**.

**Table 1:** Demographic characteristics.

Variables	Category	Non-dysphagia group (n=71)	Dysphagia group (n=72)	*P*-value
Gender, n (%)	Male	19 (26.8)	33 (45.8)	0.018
	Female	52 (73.2)	39 (54.2)	
Age (years)		51.00 [36.75, 59.00]	50.00 [37.00, 57.50]	0.336
Education, n (%)	Primary school and below	17 (23.9%)	24 (28.7)	0.518
	Junior middle school	16 (22.5)	18 (23.8)	
	Senior middle school	12 (16.9)	10 (15.4)	
	University and above	26 (36.6)	20 (32.2)	
Marital status, n (%)	Unmarried	13 (18.3)	10 (13.9)	0.847
	Divorcee	3 (4.2)	2 (2.8)	
	Married	53 (74.6)	58 (80.6)	
	Widowed	2 (2.8)	2 (2.8)	
Occupation, n (%)	Professional and technical staff	10 (14.1)	7 (9.7)	0.931
	Staff member	8 (11.3)	8 (11.1)	
	Functionary	2 (2.8)	1 (1.4)	
	Peasants	14 (19.7)	18 (25.0)	
	Workers	1 (1.4)	1 (1.4)	
	Profession	3 (4.2)	5 (6.9)	
	Others	33 (46.5)	32 (44.4)	
Smoking history, n (%)	No	63 (88.7%)	58 (80.6)	0.211
	Quit smoking	1 (1.4)	5 (6.9)	
	Yes	7 (9.9%)	9 (12.5)	
Alcohol consumption, n (%)	Never	61 (85.9)	55(76.4)	0.196
	Gave up	0 (0)	1(1.4)	
	Drink occasionally	10 (14.1)	13(18.1)	
	Drink heavily	0 (0)	3(4.2)	
Comorbidities, n (%)	No	60 (84.5)	56 (77.8)	0.304
	Yes	11 (15.5)	16 (22.2)	
Dentures, n (%)	No	64 (90.1)	63 (87.5)	0.616
	Yes	7 (9.9)	9 (12.5)	
History of choking, n (%)	No	68 (97.1)	51 (77.3)	0.000
	Yes	2 (2.9)	15 (22.7)	
Surgical site, n (%)	Bridge cerebellar corner area	53 (74.6)	55 (76.4)	0.426
	Brainstem	5 (7)	5 (6.9)	
	Encephalocele	1 (1.4)	2 (2.8)	
	Epencephal	6 (8.5)	5 (6.9)	
	Rock slope area	1 (1.4)	4 (5.6)	
	Else*	5 (7)	1 (1.4)	
Surgical duration (h)		4.35 [3.38, 6.0]	6.0 [4.5, 7.23]	0.001
Anesthesia duration (h)		6.2 [5.2, 7.43]	7.33 [6.05, 8.5]	0.004
Presence of gastric tube, n (%)	No	68 (95.8)	57 (80.3)	0.004
	Yes	3 (4.2)	14 (19.7)	
Hemoglobin		126.06 ± 17.8 2	123.32 ± 17.68	0.358
Serum albumin		38.08 ± 4.69	36.71 ± 4.2 4	0.071
Triglyceride		1.275 [0.87, 2.32]	0.980 [0.72, 1.65]	0.027
Total cholesterol		4.31 [3.77, 4.77]	3.71 [3.14, 4.44]	0.002
NRS 2002, n (%)	0	47 (66.2)	22 (30.6)	0.000
	1	15 (21.1)	25 (34.7)	
	2	5 (7)	13 (18.1)	
	3	3 (4.2)	7 (9.7)	
	4	1 (1.4)	3 (4.2)	
	5	0 (0)	1 (1.4)	
	6	0 (0)	1 (1.4)	

*Note: else: refers to the cerebellar vermis and the jugular foramen in the surgical site categories.

### 3.2 Univariate logistic regression analysis of factors influencing dysphagia

Univariate logistic regression analysis identified several risk factors for postoperative dysphagia after posterior skull base surgery. These included gender [OR: 0.432 (0.214–0.870), *P*=0.019], history of choking [OR: 10.00 (2.188–45.696), *P*=0.003], surgical duration [OR: 1.238 (1.039–1.476), *P*=0.017], anesthesia duration [OR: 1.201 (1.020–1.413), *P*=0.028], presence of gastric tube [OR: 5.567 (1.524–20.340), *P*=0.009], total score of NRS 2002 [OR: 1.948 (1.355–2.800), *P*=0.000], and total cholesterol [OR: 0.621 (0.430–0.897), *P*=0.011].

### 3.3 Multivariate logistic regression analysis of dysphagia

**Figure 2:**
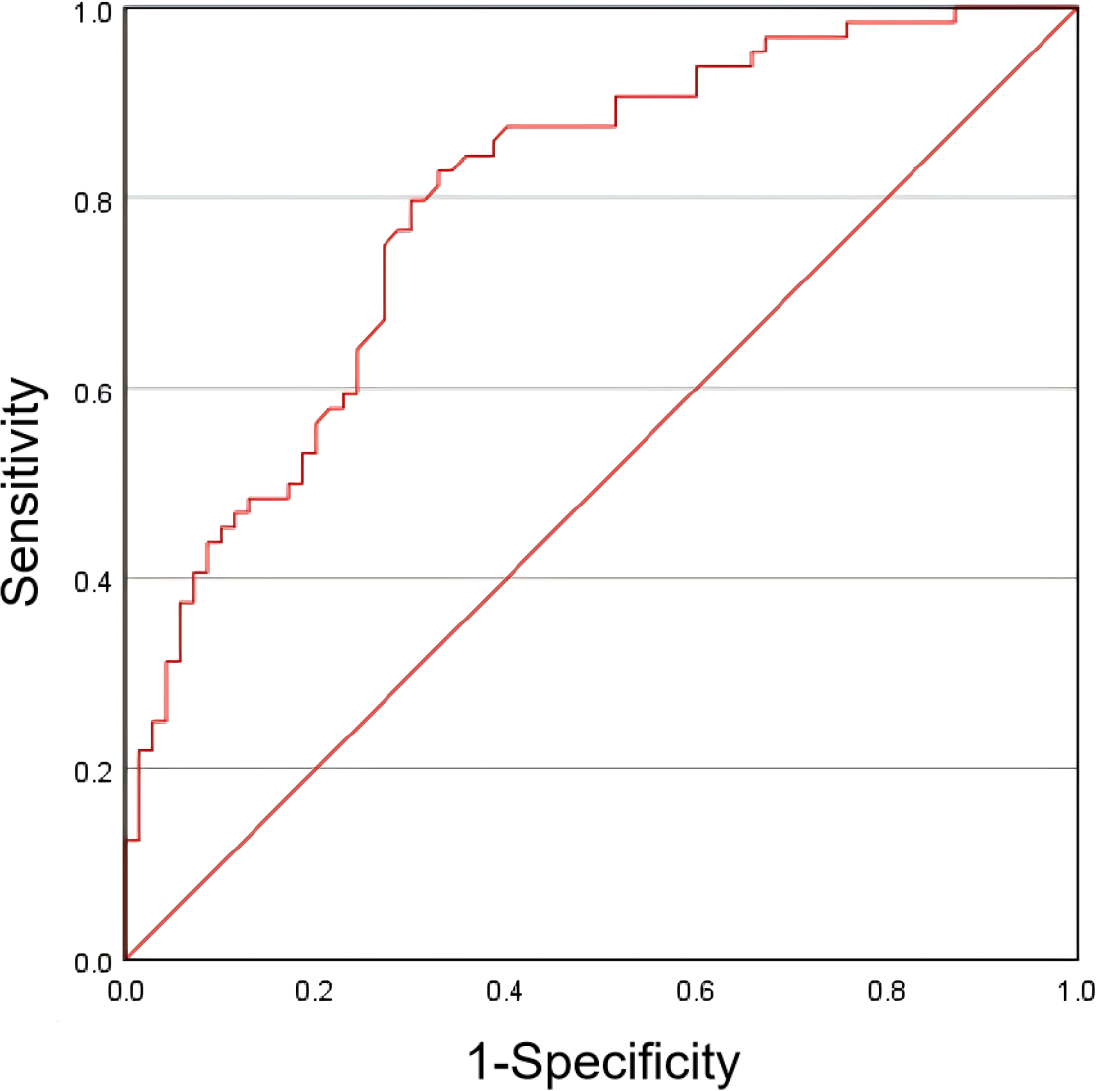
ROC curve for the diagnosis of dysphagia after posterior skull base surgery.

Prior to constructing the stepwise logistic regression model, multicollinearity among candidate predictors identified in the univariable analysis (with *P* < 0.05) was assessed. Significant collinearity was observed between surgical duration (VIF = 5.900) and anesthesia duration (VIF = 5.878). However, neither variable reached statistical significance (*P* = 0.637 and *P* = 0.725, respectively). Given the stronger clinical relevance of surgical duration in the pathogenesis of dysphagia, anesthesia duration was excluded from the final model. The final multivariate analysis revealed that history of choking, total score of NRS 2002, and surgical duration were statistically significant factors (**Table 2**). According to the Events Per Variable (EPV) criterion (18), the actual EPV was 24 (72 events ÷ 3 predictors), substantially exceeding the recommended minimum threshold (EPV ≥ 10), thereby confirming model stability. Model calibration was confirmed by the Hosmer–Lemeshow goodness-of-fit test (χ² = 9.210, df = 8, *P* = 0.325). These predictors were incorporated into a diagnostic model for dysphagia following posterior cranial base surgery. The model demonstrated a moderate level of discrimination ability, with an area under the receiver operating characteristic (ROC) curve of 0.797 (95% CI: 0.724–0.870) (**Figure 2**).

**Table 2:** Results of multivariate regression analysis of factors affecting patients with dysphagia after posterior skull base surgery.

Variables	Regression coefficient	Wald χ2	OR (95%CI)	*P*-value
Total score of NRS 2002	0.728	13.291	2.070 (1.400–3.061)	0.000
History of choking	2.446	9.120	11.548 (2.360–56.501)	0.003
Surgical duration	0.228	5.752	1.256 (1.042–1.512)	0.016

### 3.4 Results of swallowing-quality of life questionnaire

**Figure 3:**
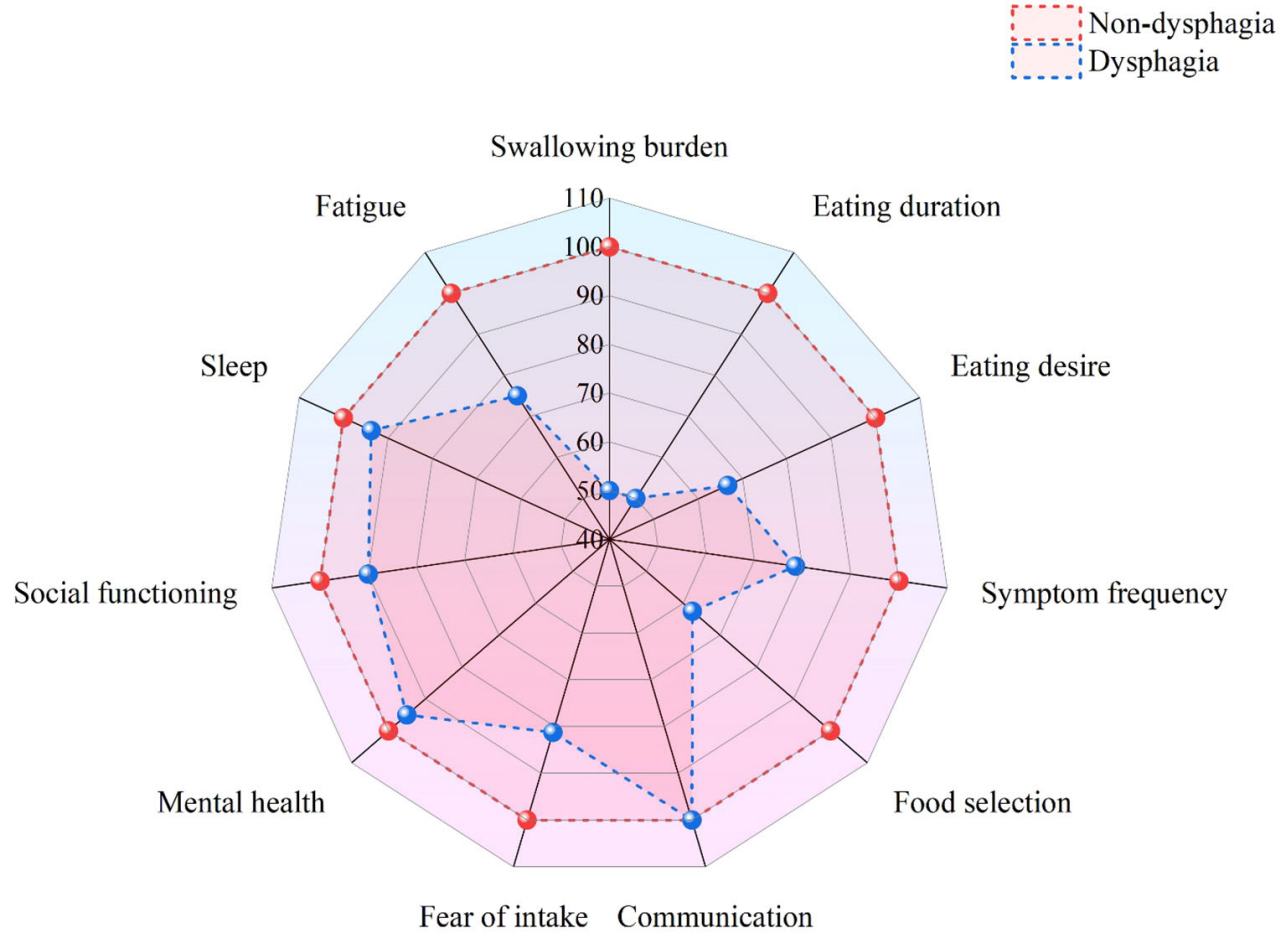
Visual comparison of SWAL-QOL scores by dimension.

Patients without dysphagia showed significantly higher total SWAL-QOL scores (92.09 ± 8.57) than those with dysphagia (68.59 ± 23.76). All domain scores were significantly lower in patients with dysphagia (*P* < 0.05), particularly in swallowing function, eating duration, and food selection (**Table 3**). The differences across various dimensions are visually presented in the radar chart (**Figure 3**).

**Table 3:** Scores on the dimensions of the swallowing-quality of life questionnaire.

Group	Dimension	Median [Q1-Q3]	Rank mean	*P*-value
Non-dysphagia	Swallowing burden	100.00 [87.50, 100.00]	94.06	<0.001
Dysphagia		50.00 [25.00, 96.88]	50.24	
Non-dysphagia	Eating duration	100.00 [75.00, 100.00]	93.58	<0.001
Dysphagia		50.00 [37.50, 84.38]	50.72	
Non-dysphagia	Eating desire	100.00 [75.00, 100.00]	87.87	<0.001
Dysphagia		66.67 [50.00, 91.67]	56.35	
Non-dysphagia	Symptom frequency	100.00 [92.86, 100.00]	93.23	<0.001
Dysphagia		78.57 [48.66, 96.43]	51.06	
Non-dysphagia	Food selection	100.00 [87.50, 100.00]	92.20	<0.001
Dysphagia		62.50 [50.00, 100.00]	52.08	
Non-dysphagia	Communication	100.00 [100.00, 100.00]	85.68	<0.001
Dysphagia		100.00 [62.50, 100.00]	58.51	
Non-dysphagia	Fear of intake	100.00 [100.00, 100.00]	90.53	<0.001
Dysphagia		81.25 [43.75, 100.00]	53.73	
Non-dysphagia	Mental health	100.00 [100.00, 100.00]	88.28	<0.001
Dysphagia		95.00 [65.00, 100.00]	55.94	
Non-dysphagia	Social functioning	100.00 [100.00, 100.00]	88.58	<0.001
Dysphagia		90.00 [55.00, 100.00]	55.65	
Non-dysphagia	Sleep	100.00 [100.00, 100.00]	83.01	<0.001
Dysphagia		93.75 [50.00, 100.00]	61.15	
Non-dysphagia	Fatigue	100.00 [50.00, 100.00]	81.75	0.004
Dysphagia		75.00 [25.00, 100.00]	62.38	

## 4 Discussion

### 4.1 High incidence of dysphagia after posterior skull base surgery

This study found that approximately 50% of patients experienced dysphagia following posterior skull base surgery. The incidence was significantly higher in males than in females (63.46% vs. 42.86%). This disparity may stem from the faster age-related decline in swallowing muscle strength (19). It should be noted that this study relied solely on the EAT-10 rather than gold-standard diagnostic tools for dysphagia, such as the videofluoroscopic swallowing study or fiberoptic endoscopic evaluation of swallowing (20, 21). This decision was based on several considerations. First, the EAT-10 has demonstrated good screening efficacy in previous validation studies (14, 16). Second, transporting patients to the imaging department during the early postoperative period poses safety risks. Finally, the EAT-10 offers advantages such as rapid implementation and low cost, which not only facilitates research but also helps reduce medical expenses for patients.

### 4.2 Risk factors for dysphagia after posterior skull base surgery

Patients with posterior skull base tumors often present with significant swallowing disorders, accompanied by clinical signs of aspiration (such as coughing or choking, nasal regurgitation after eating, and dyspnea). Previous studies have confirmed that choking was an independent predictive factor for dysphagia (22). In the multivariate logistic regression analysis of this study, the analysis showed that patients with a history of choking had a significantly increased risk of postoperative dysphagia (OR = 11.548, *P* = 0.003). Compared to other variables (NRS 2002 score, surgical duration), patients with a history of choking had the highest risk of postoperative dysphagia. Based on these findings, a history of choking was the strongest predictor of postoperative dysphagia. Healthcare providers should include a history of choking in the preoperative swallowing function assessment and guide patients in swallowing function training preoperatively to reduce postoperative complication risks. Furthermore, for patients with a positive history of choking, more careful intraoperative handling and extended postoperative monitoring time are necessary.

A study conducted by Popmann et al. showed that there was an association between dysphagia and nutritional risk (23). The results of the study indicated that the NRS 2002 score was an independent risk factor for postoperative dysphagia. For every 1-point increase in the total NRS 2002 score, the risk of dysphagia increased by 2.070 times (OR = 2.070, *P* = 0.000). The NRS 2002 score was not only a tool for quantifying nutritional risk but should also serve as a decision point for managing dysphagia during the perioperative period. Clinicians should incorporate the NRS 2002 score into routine preoperative assessments. For patients with an NRS 2002 score ≥ 3, it is recommended to consult with the nutrition department and develop a preoperative nutrition optimization plan to reduce the risk of postoperative dysphagia. Through preoperative interventions, postoperative dynamic monitoring, and multidisciplinary collaboration, this predictive indicator can be transformed into an effective tool for improving patient outcomes.

This study demonstrated that the mean operative duration in the dysphagia group was significantly prolonged (6.0 hours) compared to the non-dysphagia group (4.35 hours). The extended surgical time for dysphagia patients may be attributed to factors such as larger lesion size and more complex surgical procedures. Previous studies have confirmed a significant association between surgical duration and the incidence of postoperative dysphagia (22). Our study further quantified this relationship, with results indicating that for every additional hour of surgical time, the risk of dysphagia increased by 25.6% (OR = 1.256, *P* = 0.016). Based on these findings, it is recommended that healthcare providers include the expected surgical duration in the preoperative risk assessment system, and prioritize experienced surgical teams for complex cases to reduce surgical time. For patients whose surgical time exceeds the threshold (e.g., 6 hours), swallowing function assessments should be initiated within 24 hours postoperatively, and preventive rehabilitation training should be carried out.

Postoperative reactive brain edema typically peaks between 72 hours and 1 week, often leading to transient dysfunction due to compression of adjacent brain tissues. The most common manifestations include choking on liquids and swallowing difficulties resulting from glossopharyngeal and vagus nerve compression. Notably, swallowing function during this edematous period undergoes dynamic changes. Morgan et al. reported that 73% of children undergoing posterior skull base tumor resection exhibited dysphagia within the first 2 weeks of recovery (13). These findings emphasize the need for frequent screening of swallowing function in the early postoperative period. Longitudinal studies are also essential to track dysphagia’s progression and facilitate early identification of at-risk patients. Timely measures to address dysphagia can help prevent complications and improve prognostic outcomes (24).

However, it must be pointed out that this study has a key limitation. As a cross-sectional study, its design inherently limits our ability to infer causal relationships between the identified risk factors (such as increased nutritional risk score, prolonged operation time, etc.) and dysphagia. For instance, although decreased nutritional indicators are associated with dysphagia, it remains unclear whether dysphagia leads to insufficient nutritional intake or whether preoperative malnutrition contributes to difficulties in postoperative recovery of swallowing function. Therefore, the observed associations require further longitudinal or prospective studies to confirm causality and to gain a deeper understanding of the temporal dynamics of dysphagia in patients undergoing posterior cranial base surgery. In future studies, sequential assessments will be conducted at multiple time points, including before surgery, during the acute postoperative period (24 to 48 hours), and throughout the recovery period (one to three months), to clarify the temporal relationship and causal chain between each risk factor and swallowing dysfunction.

### 4.3 Swallowing quality of life in patients with dysphagia

Consistent with previous research findings (25), this study found that the total SWAL-QOL score of patients with dysphagia was significantly lower than that of patients without dysphagia (*P* < 0.05). In addition to being statistically significant, the difference exceeded the threshold for the minimum clinically important difference (MCID) (26). Specifically, the score differences in swallowing burden (50.00 vs. 100.00) and eating duration (50.00 vs. 100.00) reached 50 points, while the difference in food selection (62.50 vs. 100.00) reached 37.5 points, indicating substantial clinical impairment. Furthermore, 19.7% of patients with dysphagia required nasogastric tube insertion, which not only led to physiological complications, such as mucosal damage and muscle atrophy, but also imposed psychological burdens, further diminishing their quality of life (27). Based on these findings, in order to improve the quality of life for patients with dysphagia, clinical interventions should focus on the following aspects: (1) developing personalized rehabilitation plans and initiating early swallowing assessment and intervention; (2) gradually modifying dietary structure to reduce mealtime fatigue while progressively restoring normal eating patterns; (3) establishing patient support groups to provide essential psychological counseling services; and (4) forming a multidisciplinary team consisting of rehabilitation physicians, dietitians and mental health specialists to deliver integrated and holistic patient care services.

## 5 Limitations

However, this study has several limitations. Firstly, the assessment of dysphagia relied primarily on the EAT-10 score, a subjective patient-reported measure that may not fully capture the complexity or severity of dysphagia. Future research should incorporate objective assessments, such as videofluoroscopic swallow studies, to improve diagnostic accuracy. Secondly, the single-center design and modest sample size (n = 143) may limit generalizability, particularly for rare skull base pathologies. Therefore, multicenter studies with larger cohorts are needed to validate these findings and inform clinical practices. Thirdly, the lack of long-term follow-up restricted the evaluation of dysphagia recovery trajectories beyond the acute postoperative phase. Future studies should include serial evaluations (e.g., at 3, 6, and 12 months) to establish clinically actionable benchmarks for recovery. Lastly, as a cross-sectional study, this design can identify associations between risk factors and dysphagia but cannot establish causality. Longitudinal studies are required to verify the temporal sequence between identified factors and postoperative swallowing dysfunction.

## 6 Conclusions

This study identified a high prevalence of dysphagia among postoperative patients following posterior skull base surgery. Patients with dysphagia exhibited significantly lower scores across all dimensions of SWAL-QOL compared to those without dysphagia, with particularly marked impairments in swallowing burden, meating duration, and food selection. Independent risk factors for postoperative dysphagia included a history of choking, surgery duration, and the total score of NRS 2002. These findings suggest that clinical practitioners can utilize these risk factors to promptly identify high-risk patients and implement multidimensional interventions at the earliest opportunity to improve swallowing function and quality of life outcomes.

## Data Availability

The raw data supporting the conclusions of this article will be made available by the authors, without undue reservation.
